# Changes in Forcecardiography Heartbeat Morphology Induced by Cardio-Respiratory Interactions

**DOI:** 10.3390/s22239339

**Published:** 2022-11-30

**Authors:** Jessica Centracchio, Daniele Esposito, Gaetano D. Gargiulo, Emilio Andreozzi

**Affiliations:** 1 Department of Electrical Engineering and Information Technologies, University of Naples Federico II, Via Claudio 21, 80125 Napoli, Italy; 2 School of Engineering, Design and Built Environment, Western Sydney University, Penrith, NSW 2751, Australia

**Keywords:** forcecardiography, respiration, systolic time intervals, cardio-respiratory interaction, cardiac monitoring, cardio-mechanical signals, mechanocardiography

## Abstract

The cardiac function is influenced by respiration. In particular, various parameters such as cardiac time intervals and the stroke volume are modulated by respiratory activity. It has long been recognized that cardio-respiratory interactions modify the morphology of cardio-mechanical signals, e.g., phonocardiogram, seismocardiogram (SCG), and ballistocardiogram. Forcecardiography (FCG) records the weak forces induced on the chest wall by the mechanical activity of the heart and lungs and relies on specific force sensors that are capable of monitoring respiration, infrasonic cardiac vibrations, and heart sounds, all simultaneously from a single site on the chest. This study addressed the changes in FCG heartbeat morphology caused by respiration. Two respiratory-modulated parameters were considered, namely the left ventricular ejection time (LVET) and a morphological similarity index (MSi) between heartbeats. The time trends of these parameters were extracted from FCG signals and further analyzed to evaluate their consistency within the respiratory cycle in order to assess their relationship with the breathing activity. The respiratory acts were localized in the time trends of the LVET and MSi and compared with a reference respiratory signal by computing the sensitivity and positive predictive value (PPV). In addition, the agreement between the inter-breath intervals estimated from the LVET and MSi and those estimated from the reference respiratory signal was assessed via linear regression and Bland–Altman analyses. The results of this study clearly showed a tight relationship between the respiratory activity and the considered respiratory-modulated parameters. Both the LVET and MSi exhibited cyclic time trends that remarkably matched the reference respiratory signal. In addition, they achieved a very high sensitivity and PPV (LVET: 94.7% and 95.7%, respectively; MSi: 99.3% and 95.3%, respectively). The linear regression analysis reported almost unit slopes for both the LVET (R^2^ = 0.86) and MSi (R^2^ = 0.97); the Bland–Altman analysis reported a non-significant bias for both the LVET and MSi as well as limits of agreement of ±1.68 s and ±0.771 s, respectively. In summary, the results obtained were substantially in line with previous findings on SCG signals, adding to the evidence that FCG and SCG signals share a similar information content.

## 1. Introduction

The heart is often referred to as “*a pressure chamber within a pressure chamber*” because of its housing within the thoracic cavity, which is also occupied by the lungs [1,2,3]. In such a natural anatomic arrangement, the heart and lungs are forced to mechanically interact by applying variable pressures on each other, both via direct physical contact and through the blood vessels they are connected with [1,2,3,4]. The resulting cardio-respiratory interactions are known to affect various physiological parameters of the cardiac function such as the heart rate, systolic and diastolic cardiac time intervals, and stroke volume [5,6,7,8,9]. These interactions also have implications for therapy (e.g., in mechanical ventilation [1,2,10,11,12]) and are impaired by aging [13] and many cardio-pulmonary diseases [14,15,16,17]. For these reasons, monitoring both cardiac and respiratory activities is a vital task in the diagnosis and follow-up of patients as well as in patient management to help update and personalize the therapeutical approach. Unfortunately, a significant part of the physiological parameters of cardio-respiratory functions requires expensive, cumbersome, and operator-dependent instrumentation such as ultrasound imaging devices and spirometers, which do not allow for the continuous, long-term monitoring of cardio-respiratory functions.

In the last decades, there has been fervent research activity aimed at the development and validation of mechanical sensors for cardiac and respiratory monitoring [18,19,20,21,22,23,24,25]. As an example, mechanical sensors have been proposed to acquire respiratory signals, both for physiological monitoring [26,27,28,29,30,31,32,33,34] and for respiratory-gating applications in medical imaging [35,36,37,38]. Concerning the monitoring of cardiac mechanical functions, various techniques have been proposed since the late 19th century [38,39,40,41,42,43,44,45]. However, those that have survived in current research are phonocardiography [46,47], seismocardiography (SCG) [18,19,20,48,49,50,51], and ballistocardiography (BCG) [44,52,53,54,55]. SCG records the cardiac-induced accelerations of the chest wall by means of accelerometers, which have technologically evolved from bulky, heavy, metal sensors to small, lightweight, silicon sensors realized via microelectromechanical system (MEMS) technologies [18,19,20,48,49,50,51]. BCG records whole-body vibrations due to blood ejection into the vascular system and is acquired by means of a variety of sensors, weighing scales, and piezoelectric sensors [52,53,54,55]. More recently, novel techniques for cardio-respiratory monitoring have been proposed such as gyrocardiography [21,56,57] and forcecardiography (FCG) [22,23,32].

Forcecardiography is a novel cardio-mechanical monitoring technique that records the local forces induced on the chest wall by the mechanical activity of the heart and lungs [22,23,58]. Specific force sensors are employed, based on both the piezoresistive and piezoelectric effects, which are equipped with dome-shaped mechanical couplers to optimize the transduction of forces from the tissues to the sensors. Initially, FCG was acquired via piezoresistive force sensors [22], which had previously been demonstrated for muscle contraction monitoring [59], gesture recognition [60], and the control of biosignal-based human–machine interfaces [61] such as the “Federica Hand” prosthesis [62,63,64,65] and an upper-limb exoskeleton [66]. These piezoresistive FCG sensors have also been successfully used for respiratory monitoring [32]. Lately, a novel piezoelectric sensor with a specific conditioning circuit was proposed for FCG recording [23]; very recently, it was also integrated into a novel multimodal sensor for pulse wave recording from the finger [67]. This piezoelectric FCG sensor showed superior performance compared with piezoresistive FCG sensors, proving capable of capturing respiration, infrasonic cardiac vibrations, and heart sounds, all simultaneously from a single site on the chest [23]. The raw signal provided by this sensor features a large and very low-frequency component related to respiration, referred to as a forcerespirogram (FRG), and a superimposed cardiac component, which represents the actual forcecardiogram. The FRG signal captures the expansion and relaxation of the thorax during breathing and allows the accurate estimation of the inter-breath intervals and the respiratory rate, as shown in a previous study [23]. An FCG features both a sonic component (HS-FCG), corresponding to the heart sounds, and two infrasonic components, namely low-frequency FCG (LF-FCG) and high-frequency FCG (HF-FCG). HF-FCG has been shown to carry similar information to SCG [23], particularly its first derivative (dHF-FCG), which was very similar to SCG; this had a remarkably good matching of peaks and valleys, thus allowing the effective localization of aortic valve opening events and the accurate estimation of the pre-ejection period [68]. LF-FCG reflects the forces impressed on the chest wall by the emptying and filling actions of the heart chambers, thus potentially carrying information on stroke volume variations [22,23]. This low-frequency component of the precordial vibrations captured by FCG cannot be directly observed in common SCG recordings [22,23]. However, a recently proposed method based on the numerical double integration of SCG proved capable of extracting similar information from common SCG recordings [69].

It has long been recognized that cardio-mechanical signals are affected by various concurrent physiological activities, including respiration [70,71,72,73,74,75,76,77,78]. Several studies have addressed the respiratory-induced variations of heartbeat morphology in SCG, aiming either to improve the performance of ensemble averaging by applying it separately to groups of similar heartbeats or to extract respiratory signals to be used for the monitoring of the breathing activity. Pandia et al. investigated the effects of respiration on SCG signals acquired via a MEMS accelerometer, both on the mid-sternal line and the left mid-clavicular line [75], with the aim of extracting useful signals for respiratory monitoring. To this end, the authors identified three time-domain parameters that were affected by respiration; namely, the S1–S1 interval, the S1–S2 interval, and the S1–S2 intensity ratio, with S1 and S2 corresponding to the systolic and diastolic complexes of SCG in an analogy with the heart sounds terminology [75]. The S1–S1 interval is a measure of the inter-beat interval (as much as the R–R interval in ECG), so the respiratory-induced modulation of the S1–S1 interval simply reflects a physiological respiratory sinus arrhythmia [79] that is not specific to the SCG signal, but confirms its strong relationship with the cardiac cycle and its modulation due to the cardio-respiratory interplay. The S1–S2 interval is an estimate of the left ventricular ejection time [80], which is known to be modulated by respiration [5]. Respiratory-induced changes in intrathoracic pressure result in alterations to both the left and right ventricular preload, afterload, and contractility, which in turn affects the pressure gradients across the heart valves [81,82] and, eventually, the onset, duration, and force of ventricular contractions [5]. However, the authors reported that, due to the beat-to-beat changes in SCG morphology, the accurate localization of the S1 and S2 complexes to obtain the S1–S2 interval estimates was too difficult and unreliable, so they directly estimated the variation in the S1–S2 interval (ΔS1S2) by finding the time-warped version of each SCG heartbeat that exhibited the highest correlation with the previous beat, considering the related amount of time warping as an estimate of ΔS1S2. Finally, the authors suggested that the three respiratory-modulated parameters should be used together to provide a robust extraction of the respiratory activity from the SCG recordings as an alternative to relying on the near-DC components of the measured acceleration (the variable fraction of gravitational acceleration due to variable chest inclinations during breathing), which could be easily fooled by postural changes.

Azad et al. investigated the performance of six different respiratory signals comprising triaxial SCG against the respiratory volume signals extracted from respiratory air flow measurements [76]. The respiratory signals extracted from SCG were the baseline wander (information about the chest inclination), the amplitude modulation, and the frequency modulation (respiratory sinus arrhythmia). Among the SCG-derived respiratory signals, the baseline wandering emerged as the most reliable.

Taebi and Mansy addressed the effects of respiration on SCG morphology with the aim of improving the results of ensemble averaging by grouping the most similar SCG heartbeats based on the current respiratory phase or lung volume and then computing the ensemble averages separately for different groups [77]. By averaging all SCG heartbeats over several respiratory cycles, the time-varying details were filtered out of the resulting ensemble average, thus jeopardizing the effort made to separate the true underlying waveform from the noise. Taebi and Mansy proposed two grouping criteria; one based on the respiratory phase (inspiration/expiration) extracted from the respiratory airflow measurements and one based on the lung volume (low/high) obtained by integrating the respiratory airflow signals. The goodness of the grouping was assessed by comparing the differences between a group of heartbeats and their ensemble average versus the differences with the ensemble average of the other group. It transpired that grouping based on the lung volume yielded a higher goodness, thus suggesting that the lung volume is the main cause of respiratory-induced changes in SCG morphology. However, the methodology proposed by Taebi and Mansy allowed the distinguishing of only two underlying waveforms for SCG, thus neglecting the transitions between them.

Zakeri et al. developed a machine learning model based on support vector machines that was able to recognize the respiratory phase (inhalation or exhalation) by analyzing the features extracted from single SCG heartbeats [78]. The time averages of 512 intervals (4 ms in length) within each heartbeat were considered as time-domain features whilse the first 512 Fourier coefficients of each heartbeat (corresponding to the 0–500 Hz frequency band) were considered as frequency-domain features. The proposed approach yielded a recognition accuracy of about 88% in the leave-one-subject-out validation and about 95% in the single-subject validation. The rationale behind this study was to improve the performance of ensemble averaging by grouping similar SCG heartbeats in order to filter out noise and disturbances whilse maintaining the vital differences between the SCG waveforms that would have been deleted by the ensemble averaging of SCG heartbeats with different underlying morphologies.

**Figure 1:**
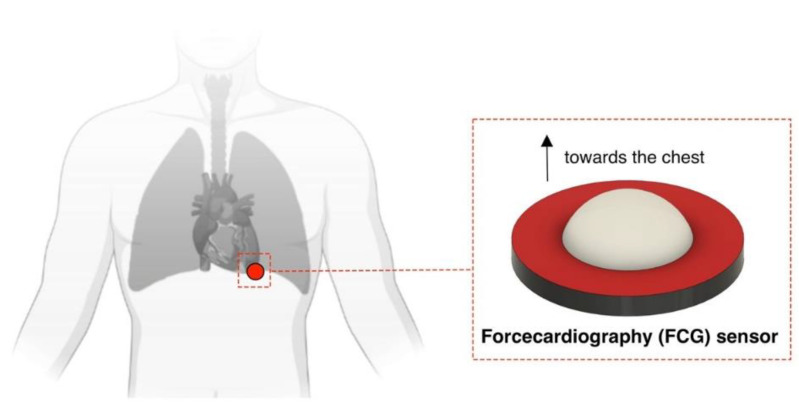
Piezoelectric FCG sensor placement on a subject. Reproduced with permission from [83].

In a recent publication [83], a retrospective analysis of the FCG signals acquired in [23] was carried out to analyze the relationship between the amplitude variations in the dHF-FCG component and the respiratory activity. In [23], six healthy volunteers were involved who were asked to stay at rest and in a seated position while breathing at a quite pace. The FCG and ECG signals were simultaneously acquired and digitized at 10 kHz with 24-bit precision. The FCG signals were acquired via a piezoelectric FCG sensor, presented precisely in [23], which had been placed on the chest of the subjects at the point of maximal impulse (see Figure 1) via medical adhesive tape and then fastened with a belt around the thorax. The retrospective analysis presented in [83] showed that respiration causes amplitude modulations of the dHF-FCG component. This finding was in agreement with the existing literature on respiratory-induced changes in SCG signals [71,73,74,75,76] and provided further evidence of the close matching between the information content of dHF-FCG and SCG signals.

This study presents a more in-depth investigation on the effects of respiration on FCG signals, focusing on the respiratory-induced changes in the morphology of dHF-FCG heartbeats. The investigation was carried out as a retrospective analysis on the same signals acquired in [23]. In particular, the beat-by-beat variation of the left ventricular ejection time (LVET) estimated from the dHF-FCG signals was considered as a first respiratory-modulated parameter. In addition, a variation in the index of the morphological similarity (MSi) between single heartbeats was considered as a second respiratory-modulated parameter. The consistency of the time trends of these parameters within the respiratory cycle was assessed by a comparison with the time trends of a reference respiration signal.

## 2. Materials and Methods

### 2.1. Signal Processing

#### 2.1.1. Pre-Processing

**Figure 2:**
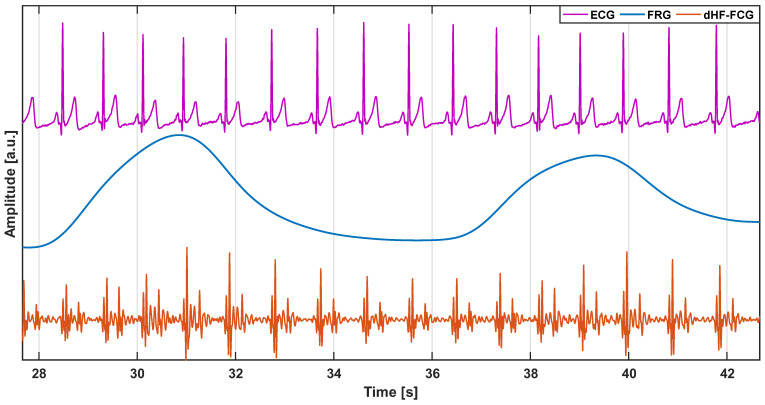
Example of FRG (blue line), dHF-FCG (orange line), and ECG (purple line) signals. The FRG signal captures the expansion and relaxation of the thorax during breathing and allows the accurate monitoring of respiration. The dHF-FCG is a high-frequency component of the FCG signal, which is very similar to the seismocardiogram and provides information about the mechanical function of the heart. The ECG is a well-established signal that provides information about the electrical function of the heart.

In a raw FCG sensor signal, a very low-frequency large component related to respiration, the FRG, appears to be superimposed on a much smaller component that reflects the cardiac activity, the actual forcecardiogram [22,23,32]. These two components must be separately analyzed to obtain information on both the breathing and cardiac activity. To this end, the FRG component was first extracted from the raw FCG sensor signal via a 3rd-order Savitzki–Golay filter with a frame length of approximately a 1.5 s time interval [84]. The FRG was then subtracted from the raw FCG sensor signal in order to isolate the actual forcecardiogram. Subsequently, the HF-FCG component was extracted from the actual forcecardiogram resulting from the respiratory signal removal via a 4th-order zero-lag Butterworth band-pass filter with cut-off frequencies of 7 and 30 Hz. Afterwards, the first derivative of the HF-FCG signal, namely dHF-FCG, was computed (as the finite forward difference) because its morphology has the highest similarity to the SCG signal, as demonstrated in [68]. Finally, the ECG signal was filtered via a 4th-order zero-lag Butterworth band-pass filter using a 0.5–40 Hz frequency band. Figure 2 shows an example of the FRG, dHF-FCG, and ECG signals. MATLAB^®^ R2018b (MathWorks, Inc., 1 Apple Hill Drive, Natick, MA, USA) was used for the signal processing.

#### 2.1.2. Extraction of the Left Ventricular Ejection Time Trends

**Figure 3:**
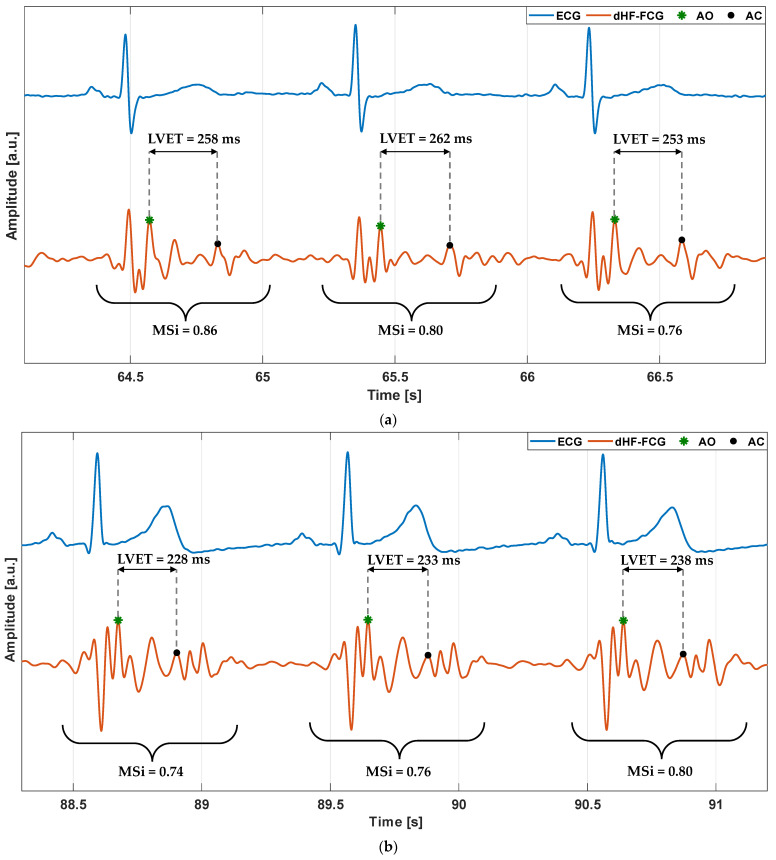
Example of LVET and MSi estimations of dHF-FCG signals (orange lines) from: (**a**) subject #2; (**b**) subject #4. ECG signals (blue lines) are also reported as a time reference for the cardiac cycle.

The left ventricular ejection time (LVET) is defined as the time interval between aortic valve opening (AO) and closure (AC) events. According to [48], the AO and AC markers were located on the dHF-FCG signal by taking advantage of a simultaneous ECG recording. Spurious AO and/or AC peaks due to portions of the signal corrupted by motion artifacts were excluded from the analysis. The LVET was then estimated for each heartbeat. Finally, a continuous LVET trend was obtained via a spline interpolation by using the MATLAB^®^ function “interp1”. Figure 3 shows an example of the AO and AC markers localization and estimation of the related LVETs for each heartbeat in some excerpts of dHF-FCG signals from subjects #2 and #4. The ECG signals that were simultaneously acquired are also reported to provide a time reference for the cardiac cycle.

#### 2.1.3. Extraction of the Morphological Similarity Index Trends

The MSi was defined with the aim of quantifying the changes in the heartbeat morphologies in the dHF-FCG signals. To this end, the main idea was to evaluate the similarity between one heartbeat, assumed to be a morphological reference, and each single heartbeat in the dHF-FCG signal. A normalized cross-correlation (NCC) was considered as the similarity index [22,23,68,69,83]. In detail, after the reference heartbeat had been selected, the R-peaks were located in the ECG signal via the Pan and Thompkins algorithm implemented in the “*BioSigKit*” MATLAB^®^ toolbox [85]; the NCC function was then computed between the reference heartbeat and the dHF-FCG signal by considering only the lags belonging to windows of 100 ms after the locations of the R-peaks. Finally, the absolute maxima of the NCC function in each window were located, providing both the location of each heartbeat in the dHF-FCG signal and a measure of its morphological similarity with the reference heartbeat. The points identified in the NCC function represented the actual values of the MSi, which were further interpolated to obtain a continuous time trend, as performed previously for the LVET. Figure 3 shows an example of the MSi estimation for each heartbeat from a few excerpts of dHF-FCG signals from subjects #2 and #4.

### 2.2. Analysis of Consistency within the Respiratory Cycle

Both the LVET and MSi trends, which were previously extracted, were filtered via a 2nd-order zero-lag Butterworth low-pass filter with a cut-off frequency of 0.5 Hz in order to filter out very small superimposed oscillations of higher frequencies. The temporal locations of the respiratory acts were then identified on both the reference signal, i.e., the FRG, and the LVET and MSi trends by considering the positive inspiratory peaks as fiducial points. Moreover, the inspiratory peaks detected on portions of the reference signal affected by motion artifacts and the corresponding peaks on the LVET and MSi trends were not considered for the analysis. Finally, inter-breath interval estimates were obtained from the three signals as the difference between the time locations of consecutive inspiratory peaks.

The sensitivity and positive predictive value (PPV) of respiratory acts detection in the LVET and MSi trends were computed according to the following mathematical expressions:

(1) $$\mathrm{Sensitivity}=\frac{\mathrm{TP}}{\mathrm{TP}+\mathrm{FN}}\;\cdot \;100$$

(2) $$\mathrm{PPV}=\frac{\mathrm{TP}}{\mathrm{TP}+\mathrm{FP}}\;\cdot \;100$$

where TP, FN, and FP indicate the number of true positives, false negatives, and false positives, respectively.

Finally, to assess the consistency of the LVET and MSi trends within the respiratory cycle, the inter-breath interval estimates obtained from these two signals were compared with those computed from the FRG signal via regression, correlation, and Bland–Altman [86,87] analyses by means of the MATLAB^®^ function “bland-altman-and-correlation-plot” [88].

## 3. Results

### 3.1. Modulation of the Left Ventricular Ejection Time Induced by Respiration

**Figure 4:**
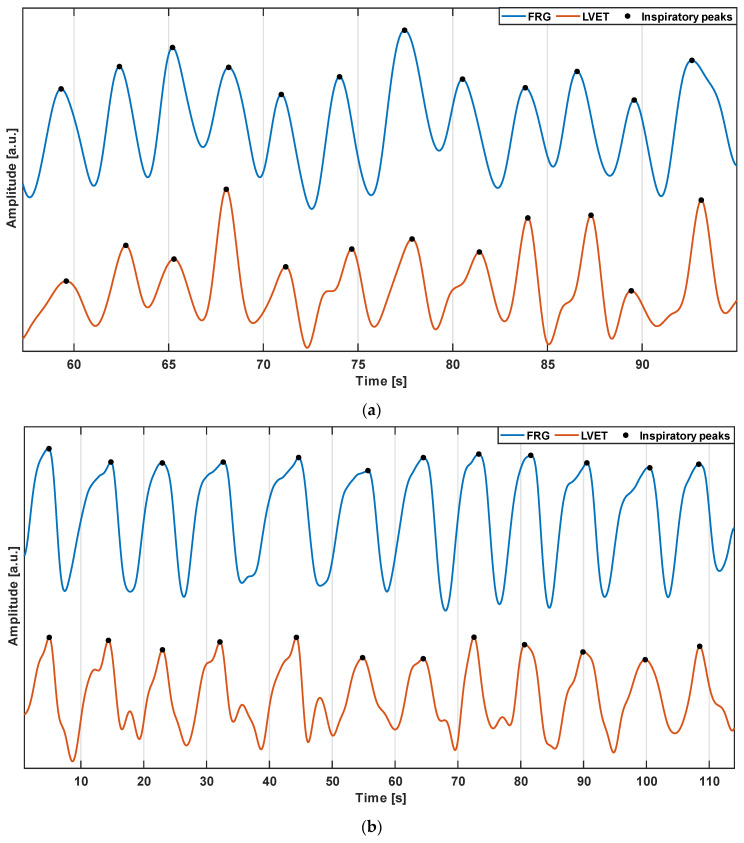
Excerpts of the FRG signal (blue line) and LVET trend (orange line) from: (**a**) subject #2; (**b**) subject #4.

Figure 4 depicts the FRG signals (blue lines) and the LVET trends (orange lines) extracted from subjects #2 and #4. The comparison showedthat the LVET, i.e., the time interval between the AO and AC markers, was modulated by respiration. The same number of respiratory acts could be detected on the two signals.

### 3.2. Modulation of the Morphological Similarity Induced by Respiration

**Figure 5:**
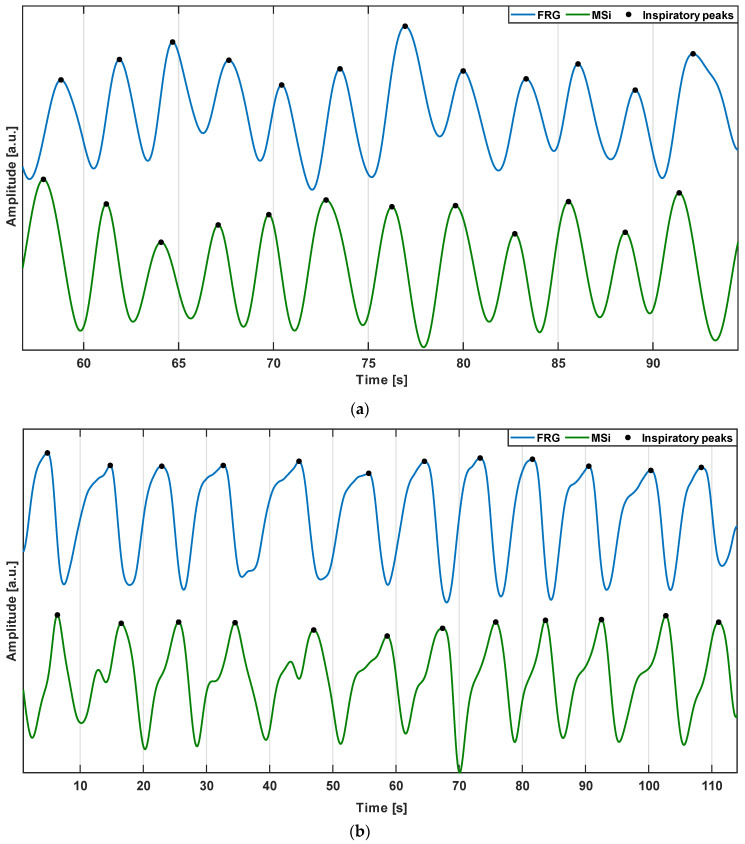
Excerpts of the FRG signal (blue line) and MSi trend (green line) from: (**a**) subject #2; (**b**) subject #4.

The FRG signals (blue lines) and MSi trends (green lines) from the same subjects #2 and #4 are depicted in Figure 5. It was observed that the MSi trend also appeared to be modulated by respiration; therefore, corresponding respiratory acts could be identified on the two signals. Moreover, the morphology exhibited a higher similarity to the respiratory signal.

### 3.3. Consistency within the Respiratory Cycle

The respiratory acts detected in the FRG signals and in the LVET and MSi trends of all subjects were considered as true positives (TP) whereas the number of missed and spurious acts, which were identified in the LVET and MSi trends with respect to the reference FRG signals, were considered as false negatives (FN) and false positives (FP), respectively. In detail, a total of 305, 289, and 303 TP were annotated for the FRG, LVET, and MSi; 16 and 2 FN as well as 13 and 15 FP were found in the LVET and MSi trends, respectively. Hence, sensitivity of 94.7% and a PPV of 95.7% were achieved for the LVET trend whereas a sensitivity of 99.3% and PPV of 95.3% were obtained for the MSi trend, as reported in Table 1.

**Figure 6:**
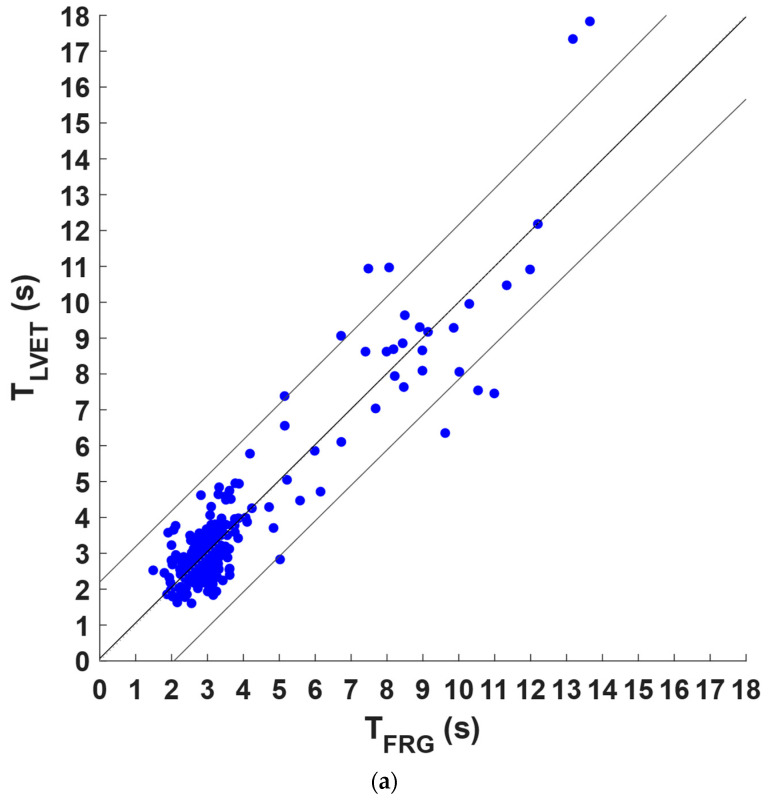

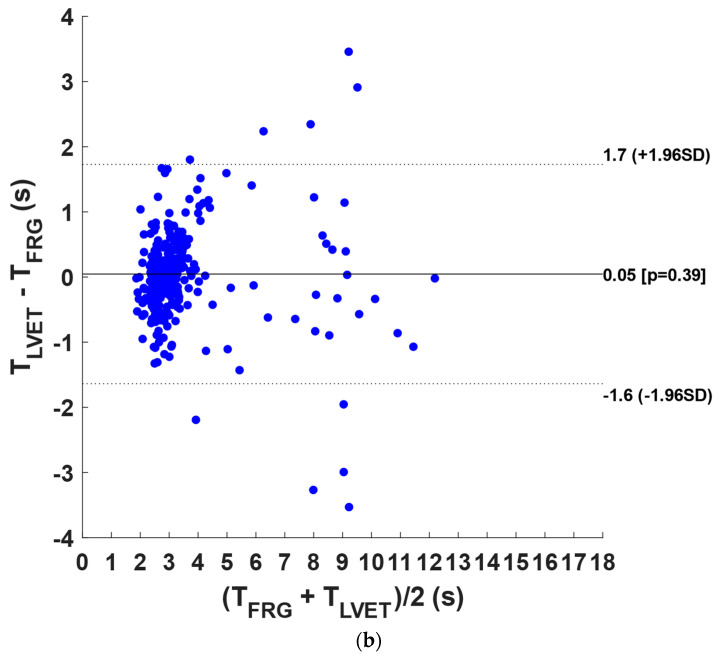
Statistical analyses on inter-breath intervals extracted from FRG and LVET time trends: (**a**) results of linear regression; (**b**) results of Bland–Altman analysis.

**Figure 7:**
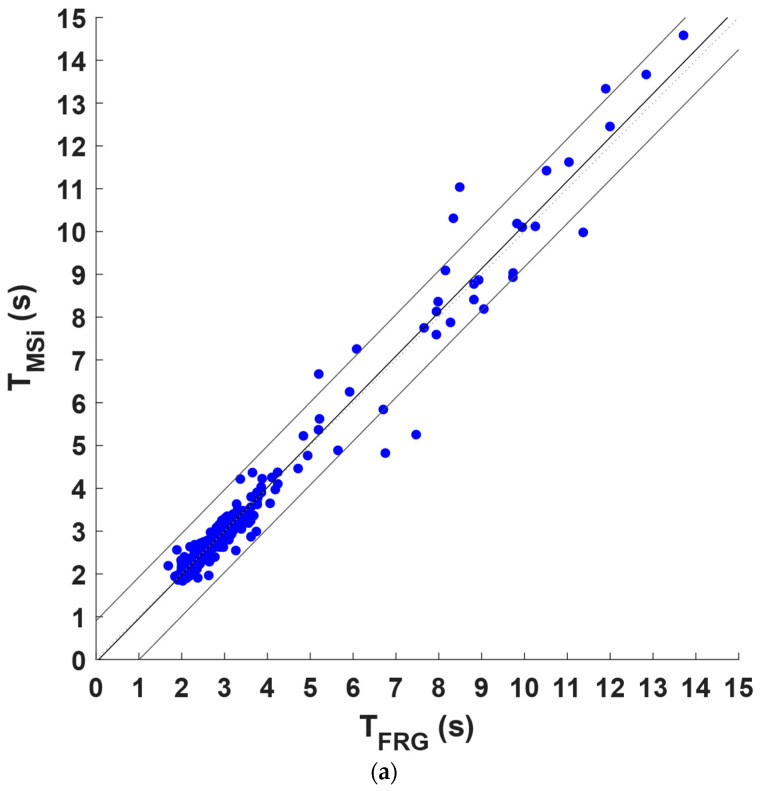

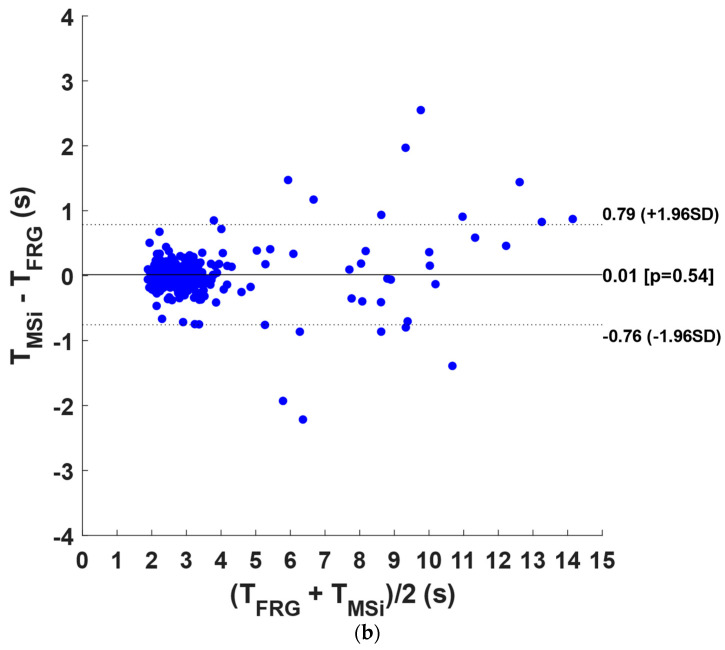
Statistical analyses on inter-breath intervals extracted from FRG and MSi time trends: (**a**) results of linear regression; (**b**) results of Bland–Altman analysis.

The inter-breath intervals obtained from the FRG signals and the LVET and MSi trends were compared via regression, correlation, and Bland–Altman analyses after excluding those related to the missed and spurious respiratory acts in the LVET and MSi trends and the corresponding intervals in the FRG signals. Statistical analyses were performed on 267 inter-breath intervals for the LVET trend and on 294 inter-breath intervals for the MSi trend; the results are depicted in Figure 6 and Figure 7, respectively. As reported in Table 2, a slope and intercept of 0.99 and 0.067 s, respectively, with an R^2^ value of 0.86 were obtained for the LVET trend as the results of the regression and correlation analyses whereas a slope and intercept of 1.02 and −0.063 s, respectively, with an R^2^ value of 0.97 resulted from the MSi trend. Moreover, the Bland–Altman analysis reported a non-significant bias for both the LVET (*p* = 0.39) and MSi (*p* = 0.54), with limits of agreement (LoA) of ±1.68 s and ±0.771 s, respectively. The differences between the measures provided by the LVET and MSi and those provided by the FRG did not exhibit clear dependences on the inter-breath intervals (see Figure 6b and Figure 7b).

## 4. Discussion

This study investigated the changes in heartbeat morphology caused in FCG signals by the respiratory activity, particularly in the dHF-FCG component, which had previously been shown to share very similar information to SCG. Specifically, two respiratory-modulated parameters of the heartbeat morphology were considered, namely the left ventricular ejection time and an index of the morphological similarity between single heartbeats. Both parameters exhibited cyclic time trends that resembled those of the reference respiratory signal (the FRG). It is worth noting that the heart rate was more than two times the respiratory rate in all the signals analyzed, which ensured that the Nyquist–Shannon criterion was always met in the estimation of the time trends of the LVET and MSi. These time trends were further analyzed in terms of consistency within the respiratory cycle in order to assess their relationship with the breathing activity. The possibility of correctly identifying the respiratory acts in the time trends of the LVET and the MSi (compared with the reference respiratory signal) was considered as a first evaluation metric, which was quantitatively determined by assessing the sensitivity and PPV of inspiratory peaks detection. Additional evaluation metrics were provided by statistical analyses of the agreement between the inter-breath intervals estimated from the inspiratory peaks previously localized in the time trends of the LVET and the MSi compared with those obtained from the FRG signal.

The preliminary results of this study clearly showed a tight relationship between the respiratory activity, as captured by the FRG, and the respiratory-modulated parameters considered in this study. A very high sensitivity and PPV were achieved for both the LVET and MSi, with the latter ensuring a higher sensitivity that was in excess of 99%. Moreover, the linear regression analysis reported almost unit slopes for both the LVET and MSi. However, the MSi achieved a remarkably higher coefficient of determination compared with the LVET (R^2^ of 0.97 versus 0.86), which suggested a higher consistency within the respiratory cycle and, therefore, a more stable modulating action engaged by respiration. This suggestion was further confirmed by the results of the Bland–Altman analysis, which reported more than halved limits of agreement for the MSi compared with the LVET.

The results obtained in this study for the LVET time trends extracted from the dHF-FCG signals were very similar to those obtained by Pandia et al. in [75] for the variations in the S1–S2 interval extracted from SCG signals. Nonetheless, they did not directly measure the S1–S2 interval, i.e., by locating specific markers on the SCG signals, because the beat-by-beat changes in morphology made the marker localization unreliable. In this study, the LVET was estimated directly by locating the time markers corresponding with the aortic valve opening and closure events (AO and AC points). This was possible thanks to the high signal-to-noise ratio of the dHF-FCG, which obviated the need for ensemble averaging to obtain reliable data [23].

The time trends of the MSi provided a global measure of the beat-by-beat changes in the heartbeat morphology. The findings of this study provide a strong suggestion that such changes are tightly linked to the respiratory activity. A similar result was presented by Taebi and Mansy in [77]. However, by dividing SCG heartbeats into only two groups (either based on the corresponding respiratory phase or lung volume), they intrinsically considered only two possible underlying morphologies for SCG, thus neglecting a type of continuum in the underlying heartbeat morphologies, which was captured, for the first time, in this study of dHF-FCG signals by analyzing the time trends of an index of the morphological similarity between heartbeats. The analysis of changes in the heartbeat morphology via the MSi could also be investigated in future for SCG signals. In summary, the results obtained from the morphological changes of heartbeats in the dHF-FCG signals, both in terms of the LVET and MSi, were substantially in line with previous findings on SCG signals, which adds to the evidence that dHF-FCG and SCG signals share the same information content.

This preliminary investigation on the respiratory-induced changes of heartbeat morphology in dHF-FCG signals was based on a retrospective analysis of data acquired during a previous study [23] and has a few limitations. The physiological signals analyzed in this study were acquired only from a small cohort of healthy subjects during quiet respiration at rest and in a sitting position. Therefore, the preliminary, yet interesting, findings of this study must be confirmed by further investigations on larger cohorts of healthy subjects and with different experimental conditions; for example, by considering different postures (e.g., supine and standing), different levels of physical activity, and a wider range of respiratory frequencies (e.g., forced respiration at very low and very high paces).

The analyses presented in this study could be extended to other components of FCG signals, e.g., the heart sounds captured in the HS-FCG, which are known to be affected by respiration [81,82], and the LF-FCG, which potentially carries information on stroke volume variations, which are also affected by respiration [1,2,3,4,5,6,7,8,9,10,11]. Considering that the morphology of cardio-mechanical signals is highly dependent on the specific sensor location over the chest [89,90], the analyses presented in this study could also be extended to multichannel measurements to be acquired by means of FCG sensor matrices.

## Figures and Tables

**Table 1:** Sensitivity and PPV of respiratory acts detection in the LVET and MSi trends.

	Sensitivity (%)	PPV (%)
LVET	94.7	95.7
MSi	99.3	95.3

**Table 2:** Results of regression, correlation, and Bland–Altman analyses for LVET and MSi trends.

	Slope	Intercept (s)	R^2^	Bias	*p*-Value	LoA (s)
LVET	0.99	0.067	0.86	Non-significant	0.39	±1.68
MSi	1.02	−0.063	0.97	Non-significant	0.54	±0.771

## Data Availability

The datasets presented in this article are not readily available because informed consent from the subjects involved was obtained only for this study and not for public availability. Requests to access the datasets should be directed to E.A. (emilio.andreozzi@unina.it).
